# Development and evaluation of human AP endonuclease inhibitors in melanoma and glioma cell lines

**DOI:** 10.1038/sj.bjc.6606058

**Published:** 2011-01-25

**Authors:** M Z Mohammed, V N Vyjayanti, C A Laughton, L V Dekker, P M Fischer, D M Wilson, R Abbotts, S Shah, P M Patel, I D Hickson, S Madhusudan

**Affiliations:** 1 Translational DNA Repair Group, Laboratory of Molecular Oncology, Academic Unit of Oncology, School of Molecular Medical Sciences, Nottingham University Hospitals, University of Nottingham, Nottingham, UK; 2 Laboratory of Molecular Gerontology, Biomedical Research Center, National Institute on Ageing, NIH, Baltimore, USA; 3 School of Pharmacy and Centre for Biomolecular Sciences, University of Nottingham, Nottingham, UK; 4 CRUK laboratories, Weatherall Institute of Molecular Medicine, University of Oxford, John Radcliffe Hospital, Oxford, UK

**Keywords:** melanoma, glioma, DNA repair, human apurinic/apyrimidinic endonuclease 1 (APE1), small molecule inhibitors

## Abstract

**Aims:**

Modulation of DNA base excision repair (BER) has the potential to enhance response to chemotherapy and improve outcomes in tumours such as melanoma and glioma. APE1, a critical protein in BER that processes potentially cytotoxic abasic sites (AP sites), is a promising new target in cancer. In the current study, we aimed to develop small molecule inhibitors of APE1 for cancer therapy.

**Methods:**

An industry-standard high throughput virtual screening strategy was adopted. The Sybyl8.0 (Tripos, St Louis, MO, USA) molecular modelling software suite was used to build inhibitor templates. Similarity searching strategies were then applied using ROCS 2.3 (Open Eye Scientific, Santa Fe, NM, USA) to extract pharmacophorically related subsets of compounds from a chemically diverse database of 2.6 million compounds. The compounds in these subsets were subjected to docking against the active site of the APE1 model, using the genetic algorithm-based programme GOLD2.7 (CCDC, Cambridge, UK). Predicted ligand poses were ranked on the basis of several scoring functions. The top virtual hits with promising pharmaceutical properties underwent detailed *in vitro* analyses using fluorescence-based APE1 cleavage assays and counter screened using endonuclease IV cleavage assays, fluorescence quenching assays and radiolabelled oligonucleotide assays. Biochemical APE1 inhibitors were then subjected to detailed cytotoxicity analyses.

**Results:**

Several specific APE1 inhibitors were isolated by this approach. The IC_50_ for APE1 inhibition ranged between 30 nM and 50 μM. We demonstrated that APE1 inhibitors lead to accumulation of AP sites in genomic DNA and potentiated the cytotoxicity of alkylating agents in melanoma and glioma cell lines.

**Conclusions:**

Our study provides evidence that APE1 is an emerging drug target and could have therapeutic application in patients with melanoma and glioma.

Monofunctional alkylating agents are routinely used for the treatment of patients with advanced melanoma and glioma. However, the response rate to chemotherapy is modest and the overall prognosis is poor. The cytotoxicity of alkylating agents is directly related to their propensity to induce genomic DNA damage. However, the ability of cancer cells to recognize this damage and initiate DNA repair is an important mechanism for therapeutic resistance that negatively impacts upon therapeutic efficacy. Pharmacological inhibition of DNA repair, therefore, has the potential to enhance the cytotoxicity of alkylating agents and improve patient outcomes (Madhusudan and Hickson, 2005; Madhusudan and Middleton, 2005).

The DNA base excision repair (BER) pathway is critically involved in the repair of bases that have been damaged by alkylating agents such as temozolomide and dacarbazine (Hoeijmakers, 2001). Although there is more than one sub-pathway of BER, in most cases base excision is initiated by a DNA glycosylase, which recognizes a damaged base and cleaves the *N*-glycosidic bond, leaving a potentially cytotoxic apurinic/apyrimidinic (AP) site intermediate (Hickson *et al*, 2000). This product is a target for the human AP endonuclease (APE1). The DNA repair domain of APE1 cleaves the phosphodiester backbone on the 5′ side of the AP site resulting in a single-strand break, which is further processed by proteins of the BER pathway. AP endonuclease 1 accounts for over 95% of the total AP endonuclease activity in human cell lines (Demple *et al*, 1991). In addition to its DNA repair activity, APE1 also performs functions such as redox regulation (mediated through a separate redox domain) and transcriptional regulation (Xanthoudakis *et al*, 1992; Okazaki *et al*, 1994; Bhakat *et al*, 2003). AP endonuclease 1 is a member of the highly conserved exonuclease III family of AP endonucleases, named after the *E. coli* homologue of APE1 (Barzilay and Hickson, 1995). The endonuclease IV family of AP endonucleases, the prototypical member of which is *E. coli* endonuclease IV (Ramotar, 1997), is structurally unrelated to APE1, despite being able to carry out the comparable AP site incision reaction (Mol *et al*, 1995; Gorman *et al*, 1997; Hosfield *et al*, 1999).

Using either antisense oligonucleotides or RNA interference approaches, several groups have reported that depletion of intracellular APE1 sensitizes mammalian cells to a variety of DNA damaging agents (Chen and Olkowski, 1994; Walker *et al*, 1994; Silber *et al*, 2002). In melanoma cell lines, APE1 downregulation led to increased apoptosis, whereas APE1 overexpression conferred protection from chemotherapy- or hydrogen peroxide-induced apoptosis. (Yang *et al*, 2005). Antisense oligonucleotides directed APE1 depletion in SNB19, a human glioma cell line lacking O(6)-methylguanine-DNA-methyltransferase, lead to potentiation of MMS and temozolomide cytotoxicity (Silber *et al*, 2002).

In patient tumours, APE1 expression may have prognostic and/or predictive significance. We have recently shown that APE1 expression has prognostic significance in ovarian, gastro-oesophageal and pancreatico-biliary cancers (Al-Attar *et al*, 2010). AP endonuclease 1 is also aberrantly expressed in other human tumours and strong nuclear expression has consistently been observed in these studies (reviewed in (Abbotts and Madhusudan, 2010)). In head and neck cancer, nuclear localisation of APE1 was associated with resistance to chemoradiotherapy and poor outcome (Koukourakis *et al*, 2001), and in cervical cancer, an inverse relationship between intrinsic radiosensitivity and levels of APE1 has been demonstrated (Herring *et al*, 1998).

Preclinical and clinical studies suggest that APE1 is a viable anti-cancer drug target. We recently initiated a drug discovery programme to identify small molecule inhibitor-lead compounds of APE1 (Madhusudan *et al*, 2005). Fluorescence-based high throughput screening of a chemical library, as well as biochemical and cellular investigations were undertaken. We reported the identification and characterisation of CRT0044876 (7-nitro-1*H*-indole-2-carboxylic acid), the first small molecule inhibitor of APE1 that potentiated the cytotoxicity of alkylating agents such as temozolomide (Madhusudan *et al*, 2005). The ability of CRT0044876 to block BER has also been demonstrated independently by other investigators (Guikema *et al*, 2007; Koll *et al*, 2008). In a recent study, BER inhibition using CRT0044876 was shown to confer selectively enhanced cytotoxicity in an acidic tumour microenvironment (Seo and Kinsella, 2009). However, the ability of CRT0044876 to block BER has not been consistently demonstrated by other groups (Fishel and Kelley, 2007) implying that further work needs to be done before a genuine lead inhibitor could emerge.

Here, we report on a new structure-based drug design strategy to identify APE1 inhibitors. This approach has allowed us to identify several novel APE1 inhibitors that potentiate the cytotoxicity of alkylating agents and that have potential as lead compounds for further optimisation and development. We also present preclinical data that support APE1 modulation as a particularly promising new strategy in melanoma and glioma where alkylating agents remain an important treatment modality.

## Materials and methods

### Enzymes, oligonucleotides and chemicals

Human APE1, uracil-DNA glycosylase and *E. coli* endonuclease IV were obtained from New England Biolabs (Ipswich, MA, USA).

The oligonucleotides; 5- F-GCCCCCXGGGGACGTACGATATCCCGCTCC-3′ and 3′-Q-CGGGGGCCCCCTGCATGCTATAGGGCGAGG-5′ (where F=fluorescein, Q=dabcyl and X=3-hydroxy-2-(hydroxymethyl)-terahydrofuran (abasic site analogue)) (Takeshita *et al*, 1987) were custom-made by Eurogentec Ltd (Southampton, UK). A uracil-containing 18-mer oligonucleotide 5′-CTCGCAAGUGGGTACCGA-3′ and its complementary oligonucleotide, 5′-TCGGTACCCGCTTGCGAG-3′ were synthesised by the Cancer Research UK central services laboratory (Clare Hall, UK). The oligonucleotides for the radiolabeled DNA substrates for HeLa whole-cell extracts (WCE) assays – 18FNMR 5′-GTCACCGTGXTACGACTC-3′ and 18GNMR 5′-GAGTCGTAGCACGGTGAC-3′ – were obtained from Trilink Biotechnologies Inc. (San Diego, CA, USA) and Midland certified reagent company, respectively.

The monofunctional alkylating agent methyl methane sulphonate (MMS) was purchased from Sigma-Aldrich (Gillingham, Dorset, UK) and dissolved in phosphate-buffered saline. Stock solutions of test compounds were dissolved in DMSO. Temozolomide (TMZ) was a gift from Dr Tracey Bradshaw, School of Pharmacy, University of Nottingham, UK. Potential APE1 inhibitors were purchased from Maybridge Chemicals (Tintagel, UK), ChemBridge Corporation (San Diego, CA, USA), ASINEX intelligent chemistry (Laan van Vredenoord, the Netherlands_)_, Life Chemicals (Braunschweig, Germany), Enamine Ltd. (Kiev, Ukraine), Specs Chemicals (Delft, the Netherlands), ChemDiv Inc. (San Diego, CA, USA), Ukrorgsynthesis Ltd (Kiev, Ukraine) and Sigma-Aldrich.

### Virtual screening strategy

Virtual screening was done against the high resolution crystal structure of APE1 (PDB accession code 1BIX). Sybyl8.0 was used to build inhibitor templates based on the previously reported APE1 inhibitor (Madhusudan *et al*, 2005) and three new pharmacophore templates designed *in silico* (M1, M2 and M3) based on the structural features of the APE1 active site (see results and discussion). Using these templates, ROCS 2.3 (Open Eye Scientific, Santa Fe, NM, USA) (Hawkins *et al*, 2007) was used to extract pharmacophorically-related (Tanimoto cut-off between 0.6 and 0.75) subsets of compounds from the ZINC database (http://zinc.docking.org/; 2008 version with *ca.* 2.6 million drug-like compounds)(Irwin and Shoichet, 2005). The 1679 filtered ligands were docked into the APE active site pocket using GOLD2.7 (Hartshorn *et al*, 2007). Predicted ligand poses were ranked on the basis of two fitness scoring functions: GOLDScore (Jones *et al*, 1997)and ChemScore (Verdonk *et al*, 2003). A total of 100 docking runs were performed for each ligand.

### Fluorescence-based AP site cleavage assay

A fluorescence-based AP site cleavage assay was performed as described previously with slight modifications (Madhusudan *et al*, 2005). Briefly, APE1 (50 nM) (New England Biolabs) was incubated in a buffer system consisting of 50 mM Tris-HCl, pH 8.0, 1 mM MgCl_2_, 50 mM NaCl, 2 mM DTT at 37°C for 10 min. 5′-F-GCCCCCXGGGGACGTACGATATCCCGCTCC-3′ and its complementary Q-labelled oligonucleotide (see above) were annealed in a buffer containing 100 *μ*M Tris-HCl, 50 mM NaCl and 1 *μ*M EDTA. AP-site cleavage was initiated by addition of the annealed substrate (25 nM) to the reaction mix. Fluorescence readings were taken at 5 min intervals during 30 min incubation at 37°C using an Envision Multilabel reader from Perkins Elmer (Cambridge, UK) with a 495 nM excitation and a 512 nM emission filter. If the DNA is cleaved at the abasic site at position 7 from the 5′-end by APE1, the 6-mer fluorescein-containing product will dissociate from its complement by thermal melting. As a result, the quenching effect of the 3′ dabcyl (which absorbs fluorescein fluorescence when in close proximity) is lost, and APE1 activity is measured indirectly as an increase in fluorescence signal (Figure 2A). Similar assays were developed for monitoring the AP endonuclease activity of endonuclease IV using a buffering system containing 10 mM HEPES-KOH, pH 7.4, 100 mM KCl and 60 ng of endonuclease IV (Trevigen, Abingdon, UK). The final DMSO concentration was maintained at 1.2% in all assays.

APE1 wild-type and D148E polymorph was quantified using NanoDrop 2000c spectrophotometer (Thermo Scientific, Wilmington, NC, USA), and 50 nM of protein was used in all assays. D148E polymorph was generated as described previously (Hadi *et al*, 2000). Experiments were repeated at least five times.

### Screening of virtual APE1 inhibitor candidates

APE1 was incubated with the candidate inhibitors at 100 *μ*M (final DMSO concentration, 1.2%) before initiating the AP site cleavage assay described in the previous section. Those candidates that showed >90% inhibition of APE1 activity were subjected to serial dilution experiments for IC_50_ calculations. In addition, screening of potential inhibitors for their specificity (at 100 *μ*M concentration) was performed using endonuclease IV cleavage assays.

### IC_50_ value estimations

To estimate IC_50_ for APE1 inhibition, the ability of the compounds to inhibit APE1 at a range of concentrations (10 nM–100 *μ*M) were evaluated in black 384-well plates. The reactions were set up as before and fluorescence intensity was measured every 5 min for 30 min following reaction initiation. Using the initial rate values from the assay, percent activity was calculated for each sample relative to a negative DMSO only control. The data was fitted to a sigmoidal dose-response model using Graphpad Prism 3.0 (GraphPad Software, La Jolla, CA, USA) and IC_50_ values were determined using the formula: % Activity=100/(1+10^(log[I]−log IC 50)^).

### Fluorescence quenching assay

To investigate the possibility that compounds might possess intrinsic quenching activity, fluorescence quenching assays were performed. Briefly, the oligonucleotides 5′-F-oligonucleotide (see above) and 3-CGGGGGCCCCCTGCATGCTATAGGGCGAGG-5′ were annealed as described previously. The double stranded oligonucleotide (5 nM) was incubated with 100 *μ*M of potential APE1 inhibitor in a buffer consisting of 50 *μ*M Tris-HCl, pH 8.0, 1 mM MgCl_2_, 50 mM NaCl and 2 mM DTT at 37°C for 30 min. Fluorescence intensity was measured every 5 min. Any hits that showed a decrease of more than 50% in the fluorescence intensity were considered as quenchers and discarded from further analyses.

### Radiolabelled oligonucleotide-based APE1 cleavage assay

This basic assay was performed as described previously (Madhusudan *et al*, 2005). Briefly, a radiolabelled uracil-containing oligonucleotide (5′-CTCGCAAGUGGGTACCGA-3′) was annealed to a complementary oligonucleotide. To generate AP sites, the annealed DNA substrate was pretreated with uracil-DNA glycosylase and the resulting AP site was chemically reduced by the addition of sodium borohydride. AP site cleavage reaction consisted of 50 nM APE1 and 0.75 ng reduced AP site double-stranded oligonucleotide incubated at 37°C for 1 h. The sample was resolved on a 15% TBE Criterion Pre Cast Gel (Bio-Rad, Hemel Hempstead, Herts, UK) and the radiolabelled substrate and reaction products were visualised using a phosphorImager (Molecular Dynamics, Buckinghamshire, UK).

### Whole-cell extract AP-site cleavage assay

HeLa cells – maintained in DMEM with 10% fetal bovine serum and 1% penicillin–Streptomycin – were harvested, washed with 1 × PBS, and the pellet was resuspended in cold 222 mM KCl plus protease inhibitors (0.5 mM PMSF, 1 *μ*g ml^−1^ each of Leupepetin and Pepstatin A), incubated on ice for 30 min, and clarified by centrifugation at 12 000 × *g* for 15 min at 4°C (Simeonov *et al*, 2009). The supernatant WCE was retained, the protein concentration determined using the Bio-Rad Bradford reagent, and aliquots were stored at −80°C. AP endonuclease activity assays using 18-mer radiolabelled oligonucleotide substrates (see above) were performed. In brief, all potential APE1 inhibitors were incubated at 100 *μ*M concentrations with 30 ng of HeLa WCE at room temperature for 15 min in incision buffer consisting of 50 mM Tris-HCl, pH 8, 1 mM MgCl_2_, 50 mM NaCl and 2 mM DTT. After incubation, 0.5 pmol ^32^P-radiolabeled THF-containing 18-mer double-stranded DNA substrate was added. Incision reactions were then carried out immediately at 37°C for 5 min in a final volume of 10 *μ*l after which the reaction was terminated by the addition of an equal volume of stop buffer (0.05% bromophenol blue and xylene cynol, 20 mM EDTA, 95% formamide), followed by denaturation of samples at 95°C for 10 min. The radiolabeled substrate and product were separated on a standard polyacrylamide-denaturing gel and quantified by phosphorimager analysis.

### Kinetics analysis

APE1 protein (80 ng) was incubated at room temperature for 30 min without or with APE1 inhibitor (5, 10 and 20 *μ*M). Fluorescent DNA substrate was then added to a final concentration of 100, 200 and 500 nM (in 40 *μ*l final volume), and enzyme activity was allowed to proceed for 30 min at 37°C. The percentage APE1 cleavage activity was plotted. Lineweaver–Burk plots and kinetic parameters (k_cat_ and K_M_) were determined from eight independent data points.

### Cell lines

MeWo, SKMel and MM418 melanoma cancer cell line were grown in RPMI culture medium (supplemented with penicillin 0.06 g l^−1^, streptomycin 0.1 g l^−1^ pH 7.0, 10% foetal bovine serum (FBS, PAA Cell Culture Company, Yeovil, Somerset, UK). U89 MG and SNB-19 glioma cell lines were grown in DMEM (supplemented with penicillin 0.06 g l^−1^, streptomycin 0.1 g l^−1^ pH 7.0, 10% foetal bovine serum (FBS, PAA Cell Culture Company). HUVEC endothelial cells were grown in a special media (199 Media+HAMS F12), and supplemented with 20% heat-inactivated foetal calf serum, 1% Hepes, 1% Glutamine, 12.5 *μ*g Human EGF, 625 ng of Human bFGF, 3750 units of Heparin, penicillin 0.06 g l^−1^, and streptomycin 0.1 g l^−1^) Only cultures with a plating efficiency of over 70% were used for analyses.

### Western blot analysis

Protein samples were prepared by lysing SKMel30, Mewo, MM418 and U89MG cells in RIPA buffer (20 mM Tris, 150 mM Nacl, 1% Nonidet p-40, 0.5% sodium deoxycholate, 1mMEDTA and 0.1% SDS) containing protease inhibitor (Sigma, Gillingham, Dorset, UK) and phosphatase inhibitor cocktail 1 and 2 (Sigma). The protein content of cleared lysates was quantified using the Bradford assay. Proteins (20 *μ*g) were separated by a 10% SDS–PAGE gel using a Tris:Glycine buffer. Following electrophoresis, proteins were transferred onto a nitrocellulose membrane and blocked by incubation with PBST (PBS, 0.05% Tween 20) containing BSA/milk. Membranes were incubated with primary antibodies (4°C/overnight, APE-1, Novus Biologicals Inc., Littleton, CO, USA 1 : 250 dilution and Actin (Abcam, Cambridge, UK) 1 : 5000 dilution) and infrared dye-labelled secondary antibodies (Li-cor, Cambridge, UK) (IRDye 800CW Donkey Anti-Rabbit IgG (H+L)) and IRDye 680CW Donkey Anti-Mouse IgG (H+L) in the dilution of 1 : 15000 for 60 min. Protein expression was determined by scanning the membranes on Licor–Odyssey's Scanner at the predefined intensity fluorescence channel (700 and 800 nm).

### Quantification of AP sites in genomic DNA

AP sites were quantified as described previously (Madhusudan *et al*, 2005). Genomic DNA was extracted from a pellet of 1 × 10^6^ cells using the guanidine/detergent lysis method. Briefly, 0.5 ml of DNAzol (Helena Biosciences, Gateshead, UK) was added to the pellet and the cell lysate was gently passed several times through a pipette. The resultant viscous solution was centrifuged at 10 000 *g* for 10 min at 25°C. DNA was precipitated from the supernatant using 0.25 ml of 100% ethanol by gently inverting the tube 5–8 times at room temperature for 1–3 min. The DNA was washed twice in 0.4 ml of 75% ethanol. The DNA was then solubilized in TE buffer (pH 8.0), and the final concentration was adjusted to 100 *μ*g ml^−1^ (using a Gene Quant *pro* spectrophotometer). AP-site determinations were performed on the genomic DNA using an aldehyde reactive probe assay kit using the protocol provided by the manufacturer (BioVision Research Products, Mountainview, CA, USA). Untreated cells were compared with cells exposed to either MMS alone, APE1 inhibitor alone or combination of MMS and APE1 inhibitor. DNA was extracted at 90 min and AP site quantified as described previously. All experiments were performed in triplicate.

### AQ_ueous_ non-radioactive cell proliferation assay (MTS assay)

To evaluate intrinsic cytotoxicity and to evaluate the potentiation of toxicity of cytotoxic agents by APE1 inhibitors, MTS assays were performed as per the manufacturer's recommendation (Promega, Southampton, UK). Briefly, 2000 cells per well (in 200 *μ*l of medium) were seeded into a 96-well plate. For HUVEC cells, 5 *μ*l of 2% type 2 gelatine (Sigma) was added to the wells and the plates were preincubated for 20 min at 37°C before seeding of cells. For intrinsic cytotoxicity assessments, cells were incubated with varying concentrations of APE1 inhibitors and the MTS assay was performed on day 5. For potentiation experiments, cells were preincubated with a relatively nontoxic concentration of APE1 inhibitor for 24 h and then exposed to MMS, temozolomide or doxorubicin. Non-radioactive cell proliferation assay was conducted as described previously.

## Results

### Virtual screening

**Figure 1:**
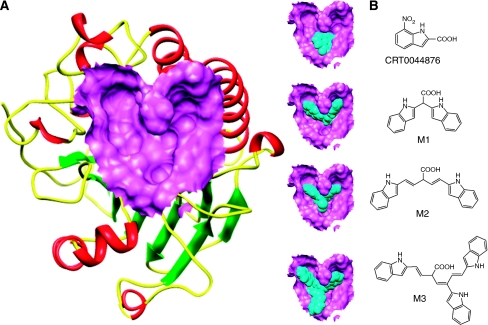
Molecular Modelling. (**A**) The DNA repair domain active site was localised on the basis of the previously reported 10 critical amino acid residues that are essential for the AP endonuclease activity of APE1 (D70, D90, E96, Y171, D210, N212, D219, D283, D308 and H309, see text for details). Visual Molecular Dynamics1.8.7 was used to visualise the crystal structure of APE1. The molecular surface in the region of the V-shaped active site cleft is shown here. (**B**) Sybyl8.0 was used to build inhibitor templates. Chemical structures and docked poses of the four prototypical ligands onto APE1 active site are shown here.

The virtual screening process requires the precise definition of the ligand-binding site in the target protein. The DNA repair domain active site was localised on the basis of the previously reported 10 critical amino acid residues that are essential for the AP endonuclease activity of APE1 (D70, D90, E96, Y171, D210, N212, D219, D283, D308 and H309) (Barzilay and Hickson, 1995; Barzilay *et al*, 1995; Rothwell and Hickson, 1996; Erzberger and Wilson, 1999; Fritz *et al*, 2003; Mundle *et al*, 2004). The active site is a well-defined deep V-shaped cleft, with a Mg^2+^ ion at its ‘elbow’ (Figure 1A).

Our virtual screening strategy was to take a known ‘first generation’ APE1 inhibitor, plus prototypical molecular scaffolds designed on the basis of the shape of the ligand-binding site, and perform a rapid structure-based similarity search of a large virtual library of drug-like molecules. ‘Hits’ from this search were then subjected to the more computationally costly process of docking-based evaluation. We used Sybyl8.0 to build molecular models for the previously reported APE1 inhibitor, CRT0044876 (Figures 1B), and to build models for three prototypical scaffolds (M1, M2 and M3) (Figures 1B) that were predicted to fit well into the APE1 binding site cleft and interact with key residues. Template M1 features a central tetrahedral centre bearing a potential Mg^2+^-interacting carboxylate group plus two heteroaromatic branches that have dimensions and relative orientations designed to fit snugly into the active site groove. Template M2 bears the same key features, but the heteroaromatic substituents are extended to interact with more of the groove . Template M3 bears an additional heteroaromatic sidechain that can access a subsidiary cleft in one branch of the ligand-binding groove (Figures 1B).

**Figure 2:**
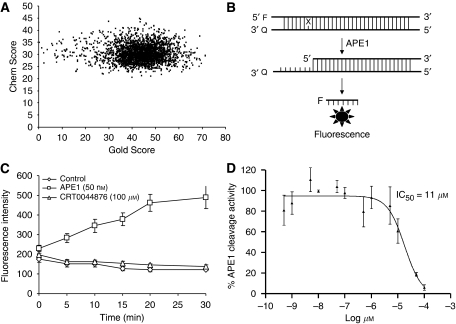
(**A**) Consensus score plot was constructed by plotting Gold Score (*x*-axis) and Chem Score (*y*-axis) for the 1679 virtual APE1 inhibitor candidates. The top ranking 25% of the compounds were shortlisted from the consensus plot and subjected to detailed biochemical analyses. (**B**) Primary screening. Fluorescence-based APE1 cleavage assay is shown here. If the DNA is cleaved at the abasic site at position 7 from the 5′ end by APE1, the 6-mer fluorescein-containing product will dissociate from its complement by thermal melting. As a result, the quenching effect of the 3′ dabcyl (which absorbs fluorescein fluorescence when in close proximity) is lost, and APE1 activity is measured indirectly as an increase in fluorescence signal. For detailed protocol see Materials and methods section. (**C**) APE1 inhibition by CRT0044876 is shown here. Control=no APE1 in reaction. (**D**) APE1 inhibition by compound 4 is shown here (IC_50_=11 *μ*M).

Using these templates, a shape-based similarity searching strategy using ROCS 2.3 (OpenEye Scientific)(Hawkins *et al*, 2007) was used to extract pharmacophorically related subsets of compounds from the ZINC database (http://zinc.docking.org/; 2008 version with *ca.* 2.6 million drug-like compounds)(Irwin and Shoichet, 2005). A total of 1679 virtual hits with similarities to the templates were identified (CRT template=359, M1 template=373, M2 template=459 and M3 template=488). The conformations of these compounds were then energy minimised and subjected to docking against the active site of the APE1 model. A consensus score plot was constructed for each virtual hit by adding the GOLDScore and ChemScore (Figure 2A). The top ranking 25% of the compounds were shortlisted from the consensus plot and subjected to detailed biochemical analyses.

### Biochemical screening

Compounds were tested in the fluorescence APE1 cleavage assay (Figure 2B). A total of 38 small molecule inhibitors of APE1 were isolated. The IC_50_ for APE1 inhibition ranged between 30 nm to 50 *μ*m. This report presents *in silico*, biochemical and cytotoxicity analyses of seven representative compounds. 5-Fluoro-1*H*-indole-2-carboxylic acid (compound 1) was originally identified using the ‘CRT0044876 (C)’ template. *N*-(3-benzooxazol-2-yl-4-hydroxy-phenyl)-2-(2-naphthyloxy)acetamide (compound 2), (3-(2-naphthyl)-5-phenyl-2,5-dihydropyrazol-1-yl]carbonylmethyl 5-nitrothiophene-2-carboxylate (compound 3), *N*-(4-fluorophenyl)-2-[4-phenylsulfonyl-2-(p-tolyl)oxazol-5-yl) sulfanyl-acetamide (compound 4) and N-(benzo(1,3)dioxol-5-ylmethyl)-4-(2-oxo-4-(thiazol-2-ylcarbamoylmethylsulfanyl)-9-thia-3,5-diazabicyclo(4.3.0) nona-4,7,10-trien-3-yl)-butanamide (compound 5) was identified through the M3 template. 2-(1H-benzoimidazol-2-ylsulfanyl)-N-((3,4 dihydroxyphenyl)methyleneamino) acetamide 1,3-bis(1,3-benzothiazol-2-ylthio)acetone (compound 6) was identified through the M2 template and 3-benzofuran-2-yl-2-benzothiazol-2-yl-3-oxo-propanenitrile (compound 7) was identified through M1 template. The chemical structures, consensus scores and biochemical profiles are summarised in Table 1. CRT0044876 was used as positive control (Figure 2C). Figure 2D demonstrates a typical APE1 inhibitory profile (compound 4, IC_50_=11 *μ*M).

**Figure 3:**
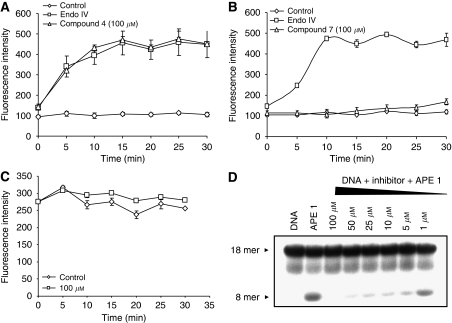
Secondary biochemical screening. (**A**) Fluorescence-based endonuclease IV cleavage assay is shown here. Compound 4 was tested at 100 *μ*M and showed no inhibition of endonuclease IV. Control=no endonuclease IV in reaction (**B**). Compound 7 was tested at 100 *μ*M and showed complete inhibition of endonuclease IV. Control=no endonuclease IV in reaction (**C**) Fluorescence queching assay did not reveal any quenching effect by compound 4. Control=no inhibitor in reaction (**D**). Radiolabelled assay showing inhibition of AP-site cleavage by APE1. Absence of 8-mer lower band indicates APE1 inhibition. See Material and methods for protcol details.

**Figure 4:**
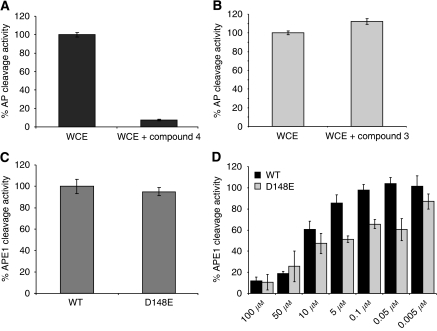
(**A**) AP endonuclease activity assays using 18-mer radiolabelled oligonucleotide substrates (see Materials and methods) were performed using HeLa whole-cell extracts (WCE). Compound 4 showed 93% inhibition of AP cleavage activity using WCE. (**B**) Compound 3, on the other hand did not inhibit WCE cleavage activity. (**C**) Testing AP site cleavage activity in wild-type and D148 polymorph. The figure shows that the activity was similar in both wild-type and the D148E polymorph. (**D**) Inhibitory activity of compound 4 against the D148E polymorphic variant of APE1 is shown here. D148E was more sensitive to inhibition by compound 4 compared with wild type.

Next, we counter-screened the compounds against endonuclease IV, an *E.coli* orthologue of APE1 that performs AP site cleavage in a way similar to APE1 but has a structurally and mechanistically different active site (Ramotar, 1997; Hosfield *et al*, 1999). We found that compounds 1-6 had no inhibitory activity against endonuclease IV (compound 4 is shown in Figure 3A), implying that these compounds are specific for APE1 and likely the exonuclease III family of AP endonucleases. Whereas compound 7 also blocked endonuclease IV activity implying non-specific activity (Figure 3B). We then tested if the compounds possessed any intrinsic fluorescence quenching activity, which was not the case (compound 4 is shown in Figure 3C). We next confirmed APE1 inhibition in a radiolabelled oligonucleotide assay (Figure 3D). To determine both selectivity and potency, the compounds were tested in a HeLa WCE assay and categorised as mild (<50% inhibition), moderate (50–75%) and strong inhibitors (>75% inhibition). Figure 4A demonstrates that Compound 4 displays strong inhibition with about 93% blockage of AP site cleavage in the WCE assay, whereas compound 3 had no activity in the WCE assay (Figure 4B).

### Inhibitory activity of compounds against the D148E polymorphic variant of APE1

The D148E polymorphic variant of APE1 has been implicated in cancer predisposition including melanoma (Li *et al*, 2006; Farkasova *et al*, 2008; Gu *et al*, 2009). In addition, the D148E polymorph may also alter ionising radiation sensitivity (Hu *et al*, 2001). We tested if our isolated inhibitors would have differential activity against the variant compared with the wild-type protein. Although the AP-site cleavage activity of D148E variant was similar to that of the wild type (Figure 4C), consistent with a previous report (Hadi *et al*, 2000), Figure 4D demonstrates that for compound 4, the IC_50_ for APE1 inhibition was significantly reduced by 50.5% for the D148E protein (5.56 *μ*M) compared with the wild type (11 *μ*M). The preferential inhibitory activity of compound 4 towards the D148E protein was also confirmed in radiolabelled oligonucleotide assays (data not shown). We were not able to demonstrate preferential inhibitory activity of other compounds either in fluorescence or radiolabelled assays.

### Kinetics analyses

**Figure 5:**
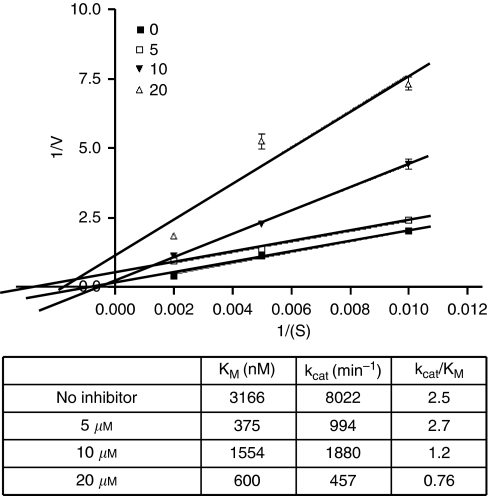
Kinetics analysis. To evaluate potential mechanism of action of APE1 inhibitor, kinetic analysis was performed. Lineweaver–Burk plots and kinetic parameters determined from eight independent data points (note: error bars are in some cases too small to see) for compound 4 is shown here. The APE1 inhibitor was tested at three dose levels (5, 10 and 20 *μ*M) and oligonucleotide substrate was evaluated at three different concentrations (100, 200 and 500 nM). The reaction was performed as described in methods. K_M_ and k_cat_ decreased at each inhibitor concentration (compared with no inhibitor) and the k_cat_/K_M_ decreased at increasing inhibitor concentration. The data is consistent with uncompetitive inhibition.

To evaluate potential mechanism of action of APE1 inhibitor, kinetic analysis was performed (Figure 5). As compound 4 had the strongest inhibitory activity (>90% inhibition) in whole-cell extracts, we selected this compound for kinetic analysis. Lineweaver–Burk plots and kinetic parameters was determined from eight independent data points. K_M_ and k_cat_ decreased at each inhibitor concentration (compared with no inhibitor) and the k_cat_/K_M_ decreased at increasing inhibitor concentration. The data is consistent with uncompetitive inhibition.

However, we cannot exclude the possibility that compound 4 operates as a weak uncompetitive inhibitor (meaning it binds the protein–DNA substrate complex), as we observed a reproducibly lower K_M_ in the presence of the compound, though this is unlikely.

### Genomic AP site accumulation in cells

**Figure 6:**
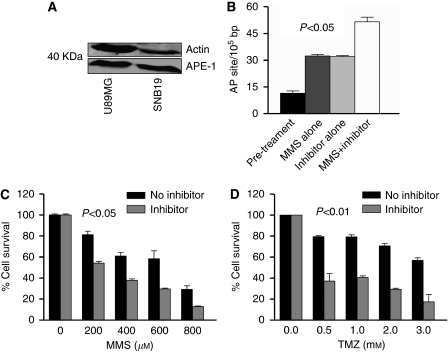
(**A**) APE1 expression in glioma cell lines is shown here. (**B**) (1 and 2). Aldehyde reactive probe assay. U89MG cells were pretreated with 10 *μ*M of compound 4 alone or MMS (600 *μ*M) or a combination of compound 4 and MMS. Genomic DNA extracted at 90 min for AP-site quatification. The combination treatment led to increased AP site content in the genomic DNA. (**C**) Inhibitor alone at 10 *μ*M was relatively nontoxic to cells (as shown in Figure 7D). We took the survival fraction as 100%. The percentage survival for those cells exposed to both inhibitor and temozolomide was plotted as a relative survival to cells exposed to the inhibitor alone. Potentiation of cytotoxicity of MMS by compound 4 (10 *μ*M) in U89 MG cell line is shown here. (**D**) Potentiation of temozolomide by compound 4 (10 *μ*M) in U89MG cell line is shown here.

**Figure 7:**
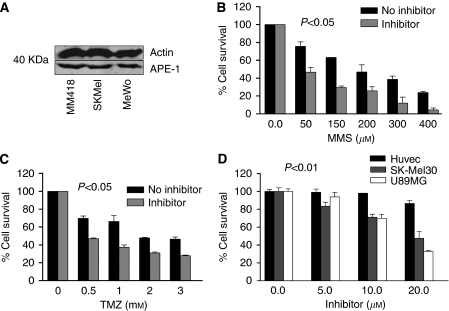
(**A**) APE1 expression in melanoma cell lines is shown here. (**B**) Inhibitor alone at 10 *μ*M was relatively nontoxic to cells (as shown in Figure 7D). We took the survival fraction as 100%. The percentage survival for those cells exposed to both inhibitor and temozolomide was plotted as a relative survival to cells exposed to the inhibitor alone. Potentiation of cytotoxicity of MMS by compound 4 (10 *μ*M) in SK-Mel30 cell line is shown here. (**C**) Potentiation of temozolomide by compound 4(10 *μ*M) in SK-Mel30 cell line is shown here. (**D**) Toxicity of compound 4 in HUVEC, SK-Mel30 and U89MG is shown here. Compound 4 was relatively nontoxic to HUVEC cells.

In order to test the biological activity of APE1 inhibitors under physiological conditions, analysis was then undertaken in melanoma cell lines (MeWo, SKMel and MM418) and glioma cell lines (U89MG and SNB-19). We initially tested if these cell lines expressed APE1 protein. Robust APE1 expression was seen in these cell lines using western blot analyses (Figures 6A and 7A). In order to confirm that the isolated inhibitors block APE1 function in living cells, the aldehyde reactive probe assay that allows quantification of genomic AP sites was utilised in this study. Figure 6B shows that compared with untreated cells, glioma cells exposed to compound 4 accumulated AP sites confirming target inhibition *in vivo*. As AP sites are obligatory intermediates during the repair of MMS-induced base damage, accumulation of AP sites were also demonstrated in cells exposed to MMS alone. Moreover, AP-site accumulation in cells exposed to a combination of APE1 inhibitor and MMS was more than the cells exposed to either agent alone. Similar accumulation of AP sites was also demonstrated in melanoma cells.

### Cytotoxicity analysis in melanoma, glioma and HUVEC endothelial cell lines

APE1 inhibitors were then tested for their inherent toxicity in melanoma and glioma cell lines. Whereas compound 1 was non-toxic (GI_50_>300 *μ*M), the GI_50_ (cell growth inhibition) ranged between 1 and 50 *μ*M for the other APE1 inhibitors: compound 2=50 *μ*M, compound 3=1 *μ*M, compound 4=17 *μ*M (glioma cell lines) and 20 *μ*M (melanoma cell lines), compound 5=14 *μ*M, compound 6=50 *μ*M and compound 7=40 *μ*M. We investigated if at relatively non-toxic concentrations, APE1 inhibitors would potentiate the cytotoxicity of monofunctional alkylating agents MMS. Figures 6C and D demonstrate that compound 4 significantly potentiated the cytotoxicity of MMS and temozolomide in U89MG glioma cell line. Similar potentiation was also demonstrated in SNB-19 glioma cell line. Figures 7B and C demonstrates the potentiation of cytotoxicity in SK-Mel30 melanoma cell line. Similar potentiation was also demonstrated in MeWo and MM418 melanoma cell lines. Potentiation of cytotoxicity was also demonstrated with other APE1 inhibitors that showed moderate to strong WCE AP-site cleavage inhibition (compound 2, 5 and 6) but not with mild WCE AP-site cleavage inhibition (compound 1). Compound 7, which was a non-specific inhibitor (i.e blocked both APE1 and endonuclease IV), did not show any potentiation of cytotoxicity and Compound 3, which was a specific APE1 inhibitor but had no activity in WCE assay, also did not shown any potentiation of cytotoxicity (data not shown).

**Figure 8:**
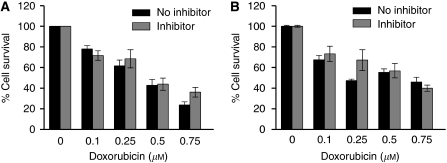
Compound 4 (10 *μ*M) did not potentiate the cytotoxicity of doxorubicin in U89MG (**A**) and SK-Mel30 (**B**) cell lines.

To exclude non-specific activity and potentiation, we performed toxic studies using doxorubicin. Compound 4 did not potentiate the cytotoxicity of doxorubicin in melanoma (SK-Mel30) and glioma cell line (U89MG) (Figures 8A and B). Similar results were seen for MeWo, MM418 and SNB-19 cells.

In order to investigate whether APE1 inhibitor was toxic to non-cancer cells, we conducted toxicity analysis in HUVEC endothelial cells. Figure 7D shows that compound 4 was relatively non-toxic to HUVECs compared with melanoma (SK-Mel30) and glioma (U89MG) cell lines. Similar results were seen for MeWo, MM418 and SNB-19 cells.

## Discussion

The overall prognosis of advanced melanoma and glioma remains poor and strategies to improve tumour response to chemotherapy remain a high priority. Blocking DNA repair may enhance cell kill in cancer and improve outcomes (Madhusudan and Hickson, 2005; Madhusudan and Middleton, 2005). APE1, a critical protein in BER, is involved in the pathogenesis of glioma and melanoma. Elevated AP endonuclease activity is frequently seen in human glioma tumours(Bobola *et al*, 2001). Moreover, in preclinical studies, antisense oligonucleotides directed APE1 depletion in SNB19, a human glioma cell line lacking O(6)-methylguanine-DNA-methyltransferase, lead to potentiation of MMS and temozolomide cytotoxicity, implying that pharmacological modulation of APE1 is a promising strategy in glioma (Silber *et al*, 2002). A recent study has demonstrated that microphthalmia-associated transcription factor (MiTF), a key transcription factor for melanocyte lineage survival, regulates APE1 expression. Microphthalmia-associated transcription factor-positive melanoma cell lines accumulated high levels of APE1 (Liu *et al*, 2009). In a separate study, downregulation of APE1 using antisense constructs promoted apoptosis in melanoma cell lines (Yang *et al*, 2005). Interestingly, the APE1 genetic polymorphism D148E may also alter melanoma predisposition (Li *et al*, 2006). These studies therefore suggest that APE1 is also a novel target in melanoma. In this investigation, we have focussed on the development of novel APE1 small molecule inhibitors and have provided the first evidence that blocking APE1 is a promising strategy in melanoma and glioma cells.

Our previous study provided the first evidence that small molecule inhibition of APE1 is a viable anticancer strategy (Madhusudan *et al*, 2005). In order to develop novel drug-like chemotypes, we recently adopted a virtual screening approach. The architecture of the active site of APE1 in the absence and presence of bound AP-DNA indicates that there is little or no remodelling of the active site upon substrate binding, a feature that is suitable for a virtual screen (Mol *et al*, 1995; Gorman *et al*, 1997). We have exploited the structural features of APE1 to develop an enhanced virtual screening strategy and identified several novel small molecule inhibitors for further drug development. Three new pharmacophore templates were designed *in silico* (M1, M2 and M3) and a total of 1679 virtual hits with similarities to the templates were identified (CRT template=359, M1 template=373, M2 template=459 and M3 template=488). Detailed biochemical screening showed that majority of the compounds conform to the M3 template, which bears an additional heteroaromatic sidechain that can access a subsidiary cleft in one branch of the ligand-binding groove (Figures 1B). Although the structural details of M3 template binding to APE1 active site is unknown, cocrytallization trials may provide structural insight to guide a rational drug-design strategy.

In this study, we also provide evidence for the first time that certain APE1 inhibitors may be more effective in blocking the endonuclease activity of the D148E polymorph (a common polymorph associated with cancer predisposition) compared with the wild type. The inability of six of the seven compounds examined to inhibit the activity of endonuclease IV provides presumptive evidence that the compounds indeed act by interaction with APE1 rather than by obscuring the abasic site on the DNA substrate. Moreover, the kinetics analysis has provided insight into the mechanism of action of the inhibitor. We have shown that compound 4 decreased K_M_, k_cat_ (compared with no inhibitor) and decreased the k_cat_/K_M_ implying uncompetitive inhibition. Future cocrytallization experiments in the presence of DNA are likely to provide further information regarding the exact mechanism of action of this compound. To assess potency and specificity of our compounds, we screened their ability to block AP-site cleavage activity using WCE. This is a good system to screen for compounds that may have non-specific binding to other cellular proteins. Compound 4 exhibited more than 90% inhibition in the WCE assays, implying strong potency and specificity. Although compound 3 blocked APE1-directed AP-site cleavage activity in purified APE1-based assay, it had no effect in the WCE assay. This implies that the compound has ‘off target’ non-specific protein-binding effect and suggests that it is unlikely to be a good development candidate.

In order to provide further preclinical evidence that blocking the repair domain of APE1 is a potential treatment strategy, we conducted studies in glioma and melanoma cell lines. We confirmed APE1 expression in these cancer cell lines. We then confirmed accumulation of AP sites *in vivo* in cells exposed to inhibitor, providing direct evidence of target inhibition *in vivo*. Intrinsic cytotoxicity for several of the inhibitors was demonstrated in glioma and melanoma cell lines, a finding consistent with the observation that APE1 downregulation in melanoma cell lines promotes apoptosis, although non-specific toxicity at higher doses of the compound cannot be excluded in our study (Yang *et al*, 2005). Interestingly, the inhibitors were relatively non-toxic to HUVEC cells implying selectivity to cancer cells. In a recent study, BER inhibition using CRT0044876 was shown to confer selectively enhanced cytotoxicity in an acidic tumour microenvironment (Seo and Kinsella, 2009), suggesting a further novel opportunity to target tumours. We then showed potentiation of MMS and temozolomide cytotoxicity in melanoma and glioma cell lines. We did not observe potentiation of doxorubicin toxicity in these cell lines implying that APE1 inhibitor potentiates chemotherapy that induce base damage and repaired through BER. Moreover, potentiation of cytotoxicity was not demonstrated in HUVEC cells, again implying selectivity to cancer cells. These studies indicate that APE1 inhibitors, either alone or in combination with chemotherapy, may be a promising strategy in cancer.

Following our initial report, other investigators have identified various APE1 inhibitors for potential pharmaceutical application (Seiple *et al*, 2008; Simeonov *et al*, 2009; Zawahir *et al*, 2009). In conclusion, these studies and our two reports (including this one), confirm the validity of APE1 as an emerging anti-cancer drug target.

## Figures and Tables

**Table 1:** Biochemical profiling of APE1 inhibitors

**APE1 inhibitors**	**Structure**	**Template**	**Score**	**APE1 (IC_50_)**	**Endo IV inhibition**	**Quencher**	**WCE Inhibition** a	**xLogP**	**Mol. Wt**
Compound 1 (5-fluoro-1H-indole-2-carboxylic acid)	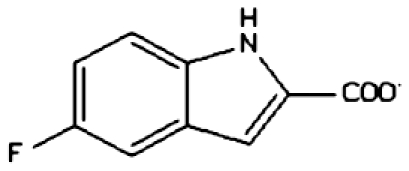	CRT	67.46	10 *μ*M	None	no	mild	1.14	178.14
Compound 2 (N-(3-benzooxazol-2-yl-4-hydroxy-phenyl)- 2-(2-naphthyloxy)acetamide)	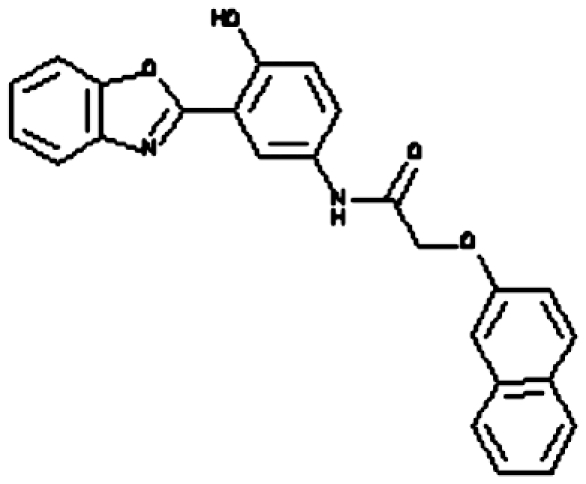	M3	98.12	25 *μ*M	None	no	Moderate	5.4	410.43
Compound 3 ((3-(2-naphthyl)-5-phenyl-2,5-dihydropyrazol-1-yl) carbonylmethyl 5-nitrothiophene-2-carboxylate)	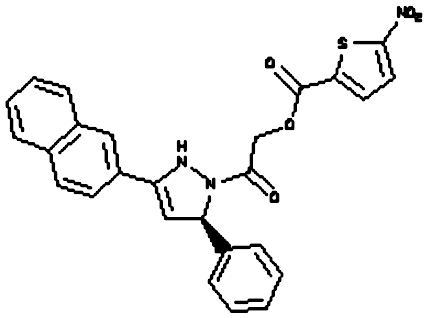	M3	81.76	3 *μ*M	None	no	None	5.33	485.52
Compound 4 (N-(4-fluorophenyl)-2-(4-phenylsulfonyl-2- (p-tolyl)oxazol-5-yl) sulfanyl-acetamide)	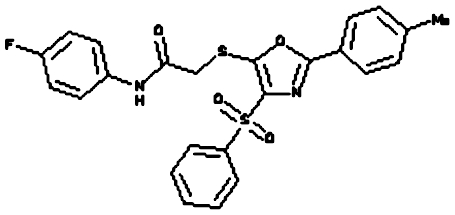	M3	82.39	11 *μ*M	None	no	strong	5.41	482.56
Compound 5 (N-(benzo(1,3) dioxol-5-ylmethyl)-4- (2-oxo-4-(thiazol-2-ylcarbamoylmethylsulfanyl)-9-thia-3, 5-diazabicyclo(4.3.0) nona-4,7,10-trien-3-yl)-butanamid	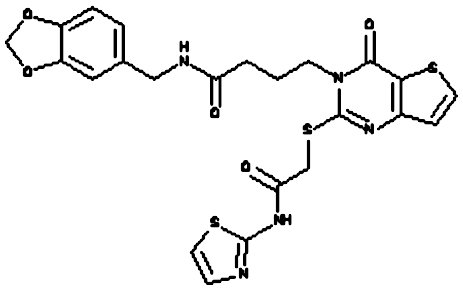	M3	83.56	12 *μ*M	None	no	Moderate	2.52	543.652
Compound 6 (1,3-bis(1,3-benzothiazol-2-ylthio)acetone)	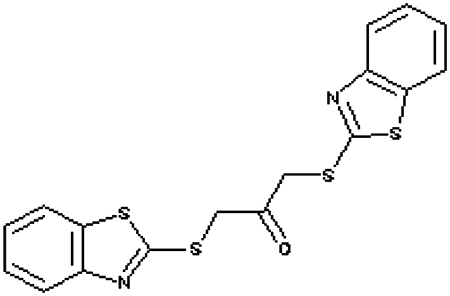	M2	81.84	3 *μ*M	None	no	Strong	5.04	388.564
Compound 7 (3-benzofuran-2-yl-2-benzothiazol-2-yl-3-oxo-propanenitrile)	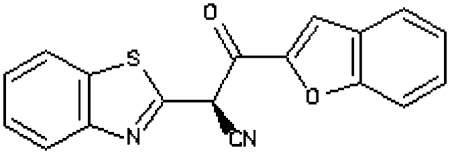	M1	93.28	2 *μ*M	yes	no	Moderate	3.57	318.357

Abbreviation: Mol. wt=molecular weight.

a Whole-cell extracts AP site cleavage inhibition: mild;<50% inhibition, Moderate; 50–75% inhibition, strong; >75%, xLogP=octanol/water partition coefficient (<5 is useful for drug likeness).
